# Molecular Dynamics Study of the Nanoindentation Behavior of Cu_64_Zr_36_/Cu Amorphous/Crystalline Nanolaminate Composites

**DOI:** 10.3390/ma14112756

**Published:** 2021-05-23

**Authors:** Wen-Ping Wu, Daniel Şopu, Jürgen Eckert

**Affiliations:** 1 Department of Engineering Mechanics, School of Civil Engineering, Wuhan University, Wuhan 430072, China; 2 State Key Laboratory of Water Resources & Hydropower Engineering Science, Wuhan University, Wuhan 430072, China; 3 Erich Schmid Institute of Materials Science, Austrian Academy of Sciences, Jahnstraße 12, A-8700 Leoben, Austria; daniel.sopu@oeaw.ac.at (D.Ş.); Juergen.Eckert@oeaw.ac.at (J.E.); 4 Institut für Materialwissenschaft, Technische Universität Darmstadt, Otto-Berndt-Straße 3, D-64287 Darmstadt, Germany; 5 Department of Materials Science, Chair of Materials Physics, Montanuniversität Leoben, Jahnstraße 12, A-8700 Leoben, Austria

**Keywords:** molecular dynamics (MD) simulation, nanoindentation, amorphous/crystalline nanolaminates (ACNLs), shear transformation zone (STZ), dislocation

## Abstract

Amorphous/crystalline nanolaminate composites have aroused extensive research interest because of their high strength and good plasticity. In this paper, the nanoindentation behavior of Cu_64_Zr_36_/Cu amorphous/crystalline nanolaminates (ACNLs) is investigated by molecular dynamics (MD) simulation while giving special attention to the plastic processes occurring at the interface. The load–displacement curves of ACNLs reveal small fluctuations associated with shear transformation zone (STZ) activation in the amorphous layer, whereas larger fluctuations associated with dislocations emission occur in the crystalline layer. During loading, local STZ activation occurs and the number of STZs increases as the indentation depth in the amorphous layer increases. These STZs are mostly located around the indenter, which correlates to the high stresses concentrated around the indenter. When the indenter penetrates the crystalline layer, dislocations emit from the interface of amorphous/crystalline, and their number increases with increasing indentation depth. During unloading, the overall number of STZs and dislocations decreases, while other new STZs and dislocations become activated. These results are discussed in terms of stress distribution, residual stresses, indentation rate and indenter radius.

## 1. Introduction

Metallic glasses (MGs) exhibit excellent mechanical, chemical and physical performances owing to the lack of grain boundaries and crystal defects [1,2,3]. However, their limited ductility with almost no global plasticity has impeded the wide application of MGs as structural materials. To overcome this limitation, amorphous/crystalline nanolaminates consisting of nanoscale amorphous and ductile crystalline layers (ACNLs) have received attention due to their high strength and good plasticity [4,5,6].

At the nanoscale, the co-deformation of glassy and crystalline layers can effectively prevent catastrophic and localized shear banding [7,8,9]. Due to the extreme difficulties of in situ experimental observation and the microstructural complexity of amorphous/crystalline interfaces, molecular dynamics (MD) simulation is suitable for probing atomic-level structural changes and the complex deformation mechanism. The STZ activation process in the amorphous layers and dislocations nucleation and dynamics in crystalline layers have recently been revealed by many MD simulations of ACNLs [10,11,12].

Previous studies [8,13,14] have demonstrated that ACNLs have both high-strength and large plastic deformability because of the special mechanical behavior at the amorphous/crystalline interfaces. Ass a popular experimental method, nanoindentation can effectively investigate the mechanical properties of materials at the nanometer scale [15,16]. Atomistic models as a useful supplement can provide some important information for the understanding complex microstructure evolution characteristics during nanoindentation; especially they have the indisputable advantage of characterizing the dislocation movement in crystalline materials [17,18,19,20] and shear bands (SB) in single-phase MGs [21,22,23,24,25,26,27], respectively. Incorporating crystalline phases mitigates the poor failure resistance in MGs, which accommodates plastic strain by dislocation slip, effectively preventing shear band propagation [4,5,6,27,28,29]. More recent simulation studies have confirmed that the crystalline phase inserted into the amorphous phase can impede the propagation of SBs, and plays a vital role in the plastic deformation mechanism of ACNLs [30,31,32,33,34,35,36,37]. Furthermore, these studies also provide insight into how the microstructure, layer thickness and individual phase properties can be tailored to optimize the mechanical properties of the composites [35,36,37,38]. Although atomic simulation of the nanoindentation behavior and the influence of different factors on mechanical properties have been considered comprehensively, the change of the local stress distribution from the amorphous layer to the crystalline layer and the deformation behavior during the unloading process were not discussed yet. Moreover, the activation of STZs, dislocation nucleation and the change of the stress distribution during nanoindentation are seldom observed in experiments, especially in the unloading process. Therefore, the details of the activation of STZs, dislocation nucleation and stress distribution characteristics during nanoindentation loading and unloading processes still remain largely speculative, and the question of how STZs and dislocations contribute to the serrated flow of ACNLs during nanoindentation is not yet fully understood. It is also important to investigate the microstructure evolution and the change of the local stress distribution during nanoindentation, which is helpful for further understanding the plastic deformation behavior and the deformation mechanism of ACNLs during nanoindentation.

In this paper, nanoindentation tests on ACNLs were performed by using MD simulations to examine the evolution of dislocation nucleation, STZ activation and stress distribution from amorphous to crystalline layers. On an atomistic level, the activation and distribution of STZs and dislocation nucleation at maximum indentation depth and after complete unloading are discussed in detail. The aim was to investigate the serrated flow behavior of ACNLs under nanoindentation and reveal the activation and evolution processes of STZs and dislocations and the deformation mechanisms during loading and unloading. The present work provides important information for understanding the nanoindentation behavior and plastic deformation mechanisms of ACNLs from an atomistic perspective.

## 2. Simulation Procedure and Stress Calculation

**Figure 1:**
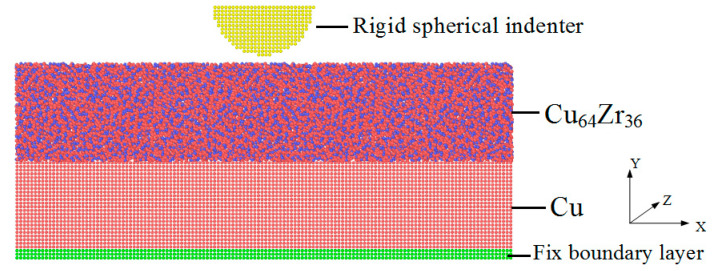
The geometries of the model.

In this work, MD simulations were carried out using an open-source LAMMPS code [38] to investigate dislocation movement, STZ activation and stress distributions for ACNLs during nanoindentation loading and unloading. The geometries of the model were shown in Figure 1. The model had a cubic orientation (i.e., *X*-[100], *Y*-[010], and *Z*-[001]), and the size of the box (*X* × *Y* × *Z*) was 250 Å × 100 Å × 250 Å. The thicknesses of the Cu_64_Zr_36_ MG and Cu crystalline layers were both 50 Å. Free boundary conditions were used on the top surface, while periodic boundary conditions (PBC) were applied in the *x* and *z* directions. The atomic positions in the last three layers (10.83 Å) at the bottom were frozen in, resembling a hard substrate. The system sizes in the directions perpendicular to the indentation direction were chosen to be large enough to avoid spurious effects of the PBC. In the present MD simulations, the modified Finnis–Sinclair-type potential for Cu–Zr binary alloys proposed by Mendelev et al. [39] were used, which had previously been successfully applied to simulate the deformation behaviors of Cu–Zr MGs and their composites [25,35,36,37,40]. The atoms in the indenter were kept fixed (i.e., the indenter was assumed to be an infinitely rigid body). At the start of the simulation, the indenter was relaxed with the conjugate gradient method at a time step of 2 fs for 20 ps to reach a minimum energy level. After relaxation, the indenter was inserted into the free surface under displacement control at an average indentation speed of *v* = 2.5 m/s to a maximum penetration depth of 85 Å. The whole indentation process was completed when the indenter returns to its initial position at the same velocity. To better highlight the material response upon mechanical loading, the initial temperature of the system was maintained at 50 K to avoid excess thermal activation [6,36,40]. Finally, the atomic configurations and the microstructural evolution were analyzed using the visualization tool OVITO [41], which provided details of the microstructural evolution of samples during nanoindentation.

The repulsive force exerted by a rigid indenter on the system in MD simulations is given by [42]:

(1) $$F(r)=K{\left(r-R\right)}^{2}$$

where r is the distance of an atom to the center of the indenter and R is the radius of the rigid indenter. Similar to previous studies, for the Cu–Zr MG system, the stiffness constant of the indenter is set to *K* = 10 eV/Å^3^ [24,43,44], and the force can be calculated by the above Equation (1).

## 3. Simulation Results and Discussion

### 3.1. Force–Displacement Curves

**Figure 2:**
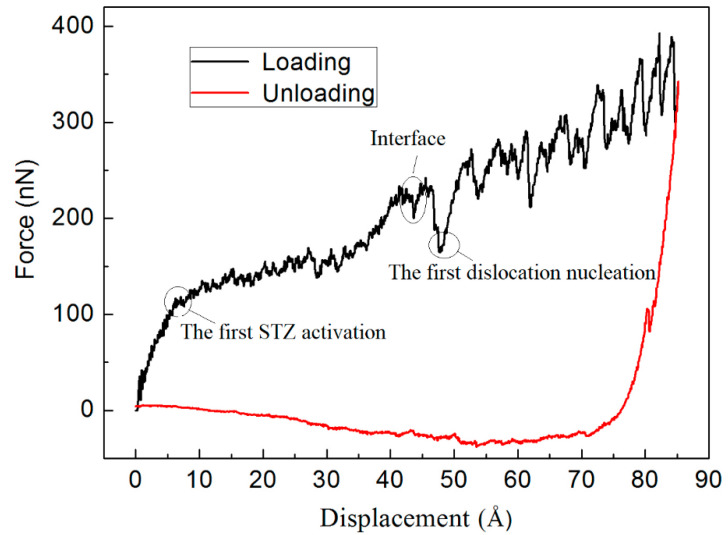
Force–displacement curve during nanoindentation loading and unloading for a loading rate of 2.5 m/s and an indenter radius of 25 Å.

Figure 2 shows the force–displacement curve for the sample with a loading rate of 2.5 m/s and an indenter radius of 25 Å during the nanoindentation loading and unloading process. The loading force increases linearly with indentation depth after the indenter tip touches the surface of the sample. Furthermore, small fluctuations were observed in the Cu_64_Zr_36_ MG layer that were related to the activation of shear transformation zones (STZs). The first STZ activation occurs when the sample enters the plastic deformation stage. At this time, the indentation depth was about 10 Å, and the corresponding atomic shear strain was about 0.2. With increasing indentation depth, the indenter reached the interface, the loading force had a relatively larger change compared with the Cu_64_Zr_36_ MG layer due to the presence of interface, and then, the indenter penetrated the Cu crystalline layer. At the same time, the observed larger fluctuations were connected with dislocation slip. The first dislocation nucleation occurred at the “pop-in” region, as shown in Figure 2. With further increasing indentation depth, the indenter penetrated the Cu crystalline layer, while the observed larger fluctuations were connected with dislocation slip. Further penetration of the indenter overcame the resistance of dislocation movement, leading to more dislocation slip. This implies that larger stresses induced plastic rearrangements and caused work hardening [32,33]. In the loading stage, the loading curves increased until reaching a maximum depth. During the unloading process, the loading force largely decreased due to the significantly large adhesion between the indenter tip and the contact region. Moreover, in the retraction part, the adhesive phenomena occur before the force returned to zero when the indenter had no impact on the substrate. These results were consistent with the results in previous studies [34,36].

**Figure 3:**
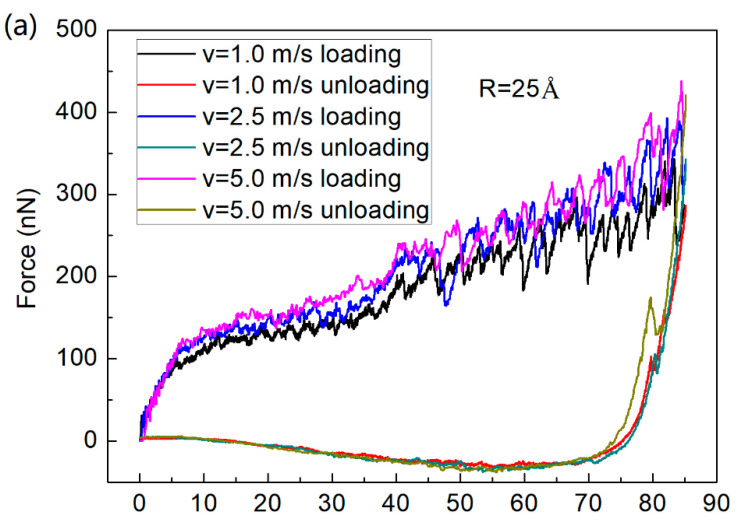

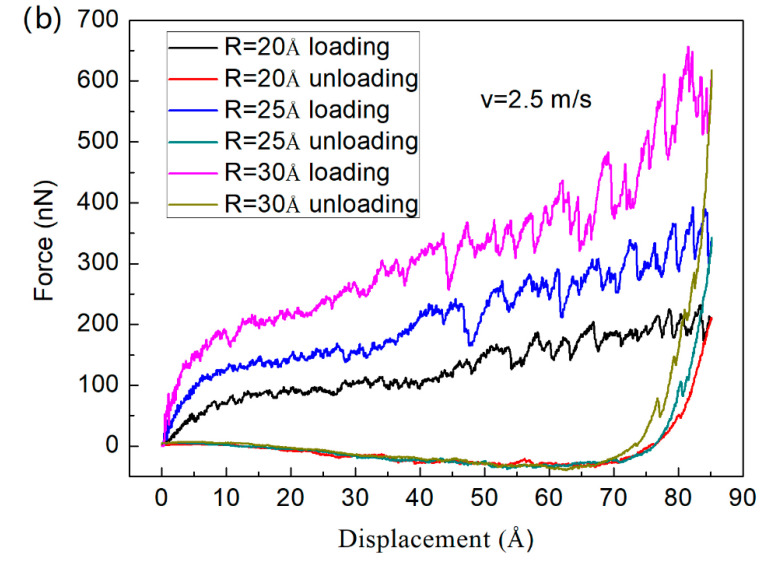
Force–displacement curves of ACNLs at different conditions. (**a**) Different loading and unloading rates, (**b**) different indenter radii.

Figure 3a,b show force–displacement curves at different loading/unloading rates and different tip radii, respectively. It can be seen that a higher force value was achieved for performing simulation at a larger loading/unloading rate because the atoms did not have enough time to release energy, resulting in higher stresses for increasing loading rates [34,36]. Moreover, for different tip radii, this was explained by the stronger structural recovery with increasing tip radius caused by the slip mechanism and dislocations [36,45].

### 3.2. Analysis of Strain/Stress Localization and Distribution

In MGs, clusters of atoms in regions with high free volume overcome the energy barrier under the action of external shear stress and undergo a synergistic shearing motion relative to the substrate, thus forming STZs [46,47,48]. As stated previously, the plastic deformation of MGs is mainly due to STZ activation and percolation processes [49], while in metallic crystals, it is due to the slip of dislocations [19]. Upon indentation, the accumulation of localization plastic events caused by shear deformation leads to STZ proliferation under the indenter and eventually forms the shear deformation region (SDR) [36,37,50,51,52,53].

**Figure 4:**
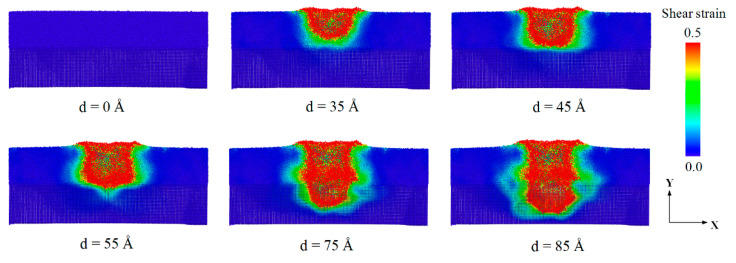
Cross-section snapshots of atomic shear strain distributions of ACNLs during nanoindentation loading.

Here, the local atomic shear strain for each atom was calculated for quantifying plastic deformation at the atomic level, and atoms with a shear strain larger than 0.2 were considered to visualize the local plastic deformation within the sample [36,43,54]. For visualization, a color code of the shear strain between 0 and 0.5 was generally used [36,44,54]. Figure 4 shows the shear strain distribution of Cu_64_Zr_36_/Cu amorphous/crystalline nanolaminates (ACNLs) at different indentation depths. In the simulation, the radius of the indenter was 25 Å, and the loading rate was 2.5 m/s. All the atoms are colored according to the shear strain value during the nanoindentation loading, where the red and blue atoms represent higher and lower shear strain, respectively. The atomic shear strain distribution is shown at different indentation depths in the range of 0 to 85 Å. The zones of high shear strain or STZs were concentrated around the indenter, and the number of atoms with high shear strain value increased with the increase of the indentation depth. When the indenter was pressed into the Cu_64_Zr_36_ amorphous layer, STZs activate at “weak spots” (defined as regions with large free volume) around the contact zones, and the SDR emerged around the indenter [55]. The shear strain was further increased at an indentation depth of 35 Å, and STZs were generated at the periphery of the previous SDR, resulting in a further expansion of the SDR. As the indentation depth increased to 45 Å, the indenter was still in the amorphous layer. The SDR extended further outwards.

**Figure 5:**
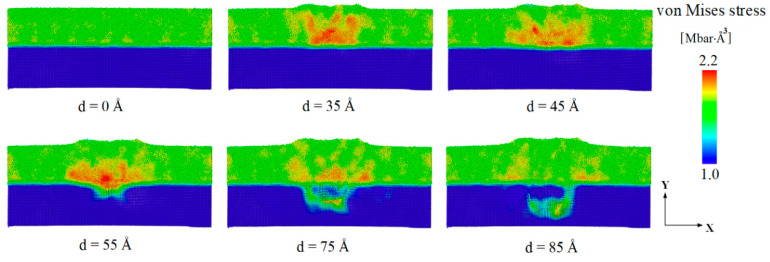
Cross-section snapshots of von Mises stress distributions of ACNLs during nanoindentation loading.

Similar to the distribution of the shear strain in Figure 4, the von Mises stress distribution also showed obvious localization; higher von Mises stress regions represent clusters of atoms with the plastic flow, and the STZs gather from the local areas of these plastic flow clusters. Figure 5 shows the von Mises stress distribution of the Cu_64_Zr_36_/Cu nanolaminates during indentation loading. The high von Mises stress was also concentrated around the indenter more than at other locations of the Cu_64_Zr_36_ amorphous layer. Moreover, the local stress increases by the pressure of the indenter with increasing indentation depth. When the indenter penetrated the Cu crystal layer, the high von Mises stress was gradually dispersed in the Cu_64_Zr_36_ amorphous layer, but the local stress was still concentrated around the indenter in the crystalline layer. The reason was that the surface of the substrate was directly affected by the pressure of the indenter; the local stress of the atomic zone around the indenter was higher than in other areas. In brief, the high von Mises stress concentration zones were concentrated around the indenter during nanoindentation loading. The local stress in the amorphous layer was larger than in the crystalline layer, which was consistent with findings by Goryaeva et al. [56]. This also indicated that the ACNLs with incorporated crystalline layers, which dissipated energy and accommodated plastic strain by dislocation slip, could avoid catastrophic localization, effectively impeding SB propagation.

### 3.3. Dislocation Analysis

**Figure 6:**
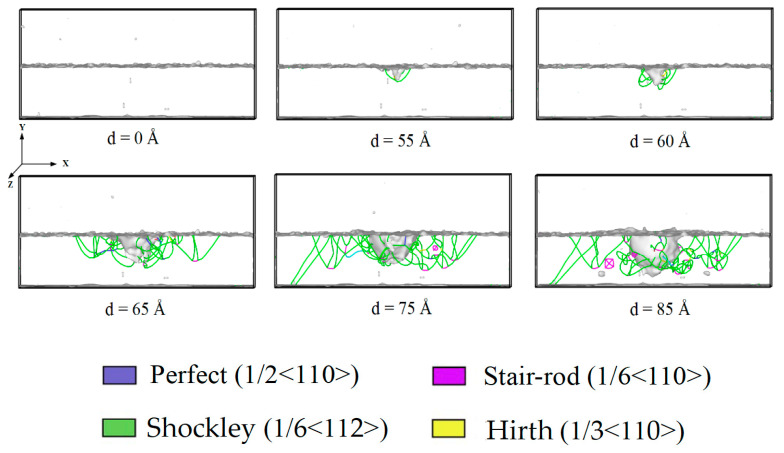
The process of dislocation motion ofACNLs during loading.

**Figure 7:**
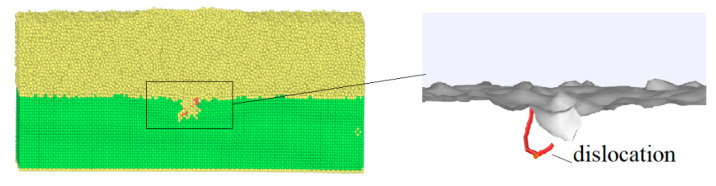
The dislocation emits from the interface of ACNLsat indentation depth of 50 Å.

In the amorphous layer, the high shear strain first appeared along the sides of the indenter because the pressure of the indenter induced deformation (see Figure 6). With further penetration of the amorphous layer, larger plastic zones formed around the indenter, but there was no nucleation of dislocations or stacking faults up to an indentation depth of 45 Å. Hence, none of these plastic zones were a potential origin of dislocation nucleation in the crystalline layer, as previously reported in the literature [32,33,34,35]. One reason for that could be that in our case, no dominant shear band forms; thus, the local stress fluctuations were too small to generate dislocation slip. When the indentation depth increased to 50 Å, the indenter penetrated the crystalline layer; dislocation emitted from the interface between the amorphous and crystalline layers, expressed through the appearance of dislocation in the crystalline layer, as shown in Figure 7. As the indentation depth increased from 55 to 85 Å, the number of slip dislocations increased. These dislocations mainly included 1/2〈110〉 perfect dislocations, 1/6〈112〉 Shockley partial dislocations, 1/6〈110〉 stair–rod partial dislocations, and 1/3〈100〉 Hirth dislocations. The plastic deformation of the crystalline layers was much smaller than that of the amorphous layers. This was consistent with the local stress distribution in the amorphous layers being much larger than in the crystalline layers. As the indentation depth increased, the plastic deformation slightly increased in the crystalline layers. The plastic deformation was significantly localized and mainly concentrated under the indenter.

### 3.4. Analysis of STZs and Dislocations during Loading and Unloading

**Figure 8:**
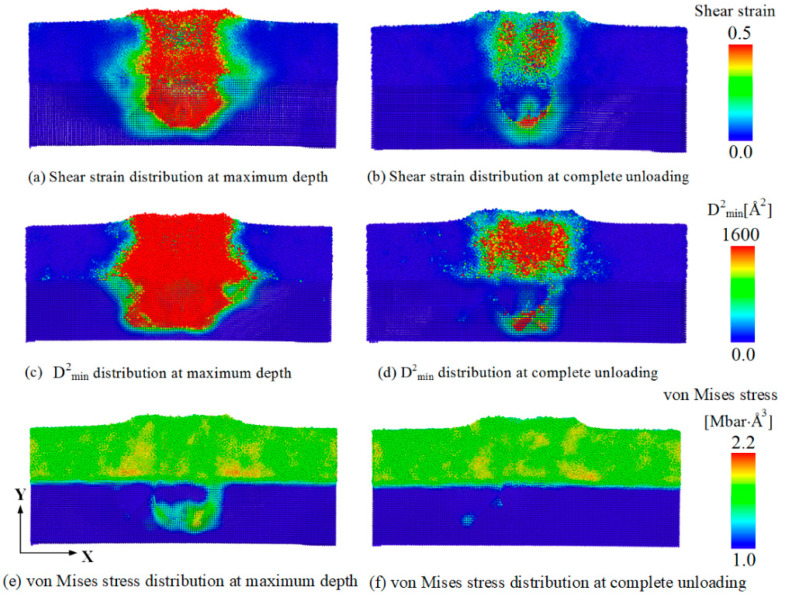
Cross-section snapshots of atomic shear strain, D^2^_min_, and von Mises stress of ACNLs at maximum depth and complete unloading stages. (**a**,**c**,**e**) are atomic shear strain, D^2^_min_, and von Mises stress of ACNLs at maximum depth stage, respectively; (**b**,**d**,**f**) are atomic shear strain, D^2^_min_, and von Mises stress of ACNLs at complete unloading stage, respectively.

**Figure 9:**
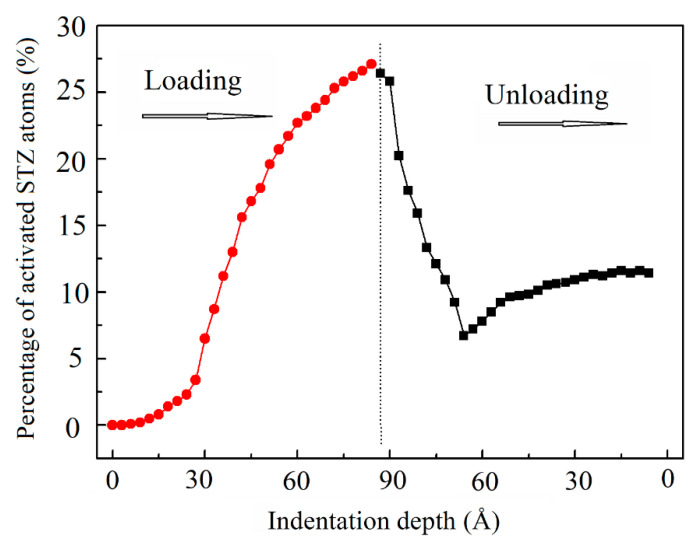
Variation of the percentage of activated STZ atoms (those atoms with shear strains > 0.2) with indentation depth during loading and unloading.

Atomic-scale deformation is often characterized by the local shear invariant [27] or the non-affine squared displacement (D^2^_min_) as a structural parameter providing information about non-elastic deformations [55]. To elucidate the activation of STZs and dislocation movement during nanoindentation loading and unloading, we calculated the atomic shear strain, D^2^_min_, and the von Mises stress at maximum indentation depth and after complete unloading stages. Figure 8 shows cross-section snapshots of atomic shear strain, D^2^_min_, and von Mises stress of nanolaminates at maximum depth and complete unloading stages. The distributions of atomic shear strain and D^2^_min_ reveal that high shear strain and high D^2^_min_ concentration zones developed around the indenter at the maximum indentation. The high shear strain and high D^2^_min_ concentration zones were still concentrated around the indenter after complete unloading. A high von Mises stress was also concentrated around the indenter at the maximum indentation stage. Moreover, the von Mises stress was dispersed in the Cu_64_Zr_36_ amorphous layer, but high local stress was still concentrated around the indenter even after complete unloading. Figure 8 quantitatively presents the variation of the percentage of STZ atoms with indentation depth during the loading and unloading stages. To quantitatively demonstrate the changes in the STZ activation during the loading and unloading process, as mentioned above, atoms with a shear strain larger than 0.2 were considered to visualize the local plastic deformation within the sample [36,42,53], which was also consistent with our previous study on the value of atomic shear strain of STZ activation during creep [57]. The percentage of STZ atoms in the total number of atoms increases slowly from 0% to 4.5% when the indentation depth increases from 0 to 30 Å, and the percentage of STZs shows a very rapid increase from 4.5% to 27.0% at indentation depths from 30 to 85 Å (maximum depth). During the unloading stage, the percentage of STZ atoms very rapidly decreases from 27.0% to 7.0%. Subsequently, the percentage of STZ shows a small increase and basically maintains at 9.0% until complete unloading, as shown in Figure 9. This indicated that some new STZs still activate in the unloading stage, and these activated STZs were mainly concentrated around the indenter both at the loading and unloading stages.

**Figure 10:**
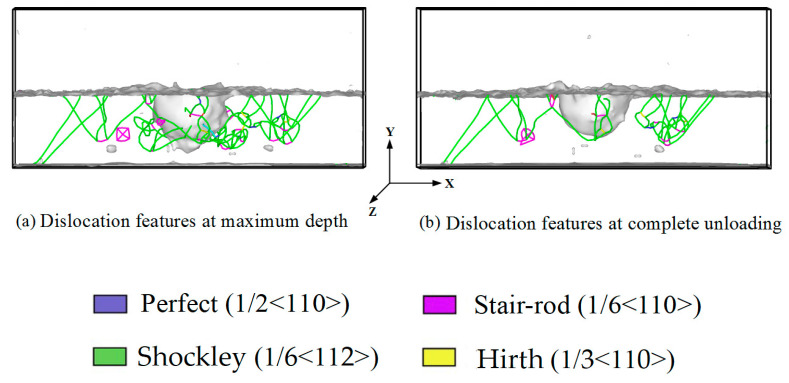
The process of dislocation motion at maximum depth and complete unloading.

**Figure 11:**
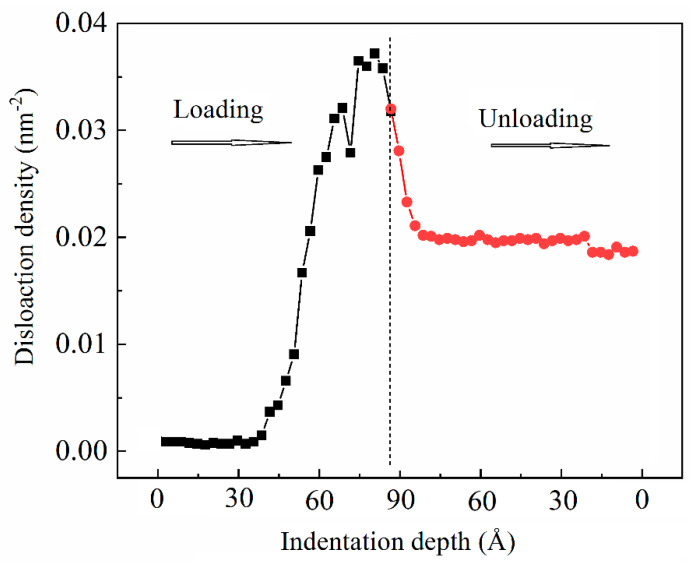
Variation of the dislocation density with indentation depth during loading and unloading.

Figure 10 shows the dislocation evolution in the nanolaminates at maximum depth and complete unloading stated. It could be clearly seen that a larger number of dislocations emits from the interface in the crystalline layer; these dislocations were mainly composed of 1/6〈112〉 Shockley partial dislocations and 1/6〈110〉 stair–rod partial dislocations. The number of dislocations decreases, but some dislocations survive the unloading process. Figure 11 shows the variation of the dislocation density with indentation depth during loading and unloading. During loading, the dislocation density was close to zero in the amorphous layer, indicating no dislocations nucleated when the indenter was pressed into the amorphous layer. Once the indenter penetrated the crystalline layer, dislocations were emitted from the interface, and their number increases rapidly with increasing indentation depth. The dislocation density decreases rapidly during unloading, and when the indenter returns to the initial position, the dislocation density remains unchanged. This indicated that some dislocations still existed in the crystalline layer even after complete unloading. These results were consistent with those in Figure 9.

## 4. Conclusions

MD simulations were performed to investigate the nanoindentation behavior of Cu_64_Zr_36_/Cu amorphous/crystalline nanolaminate composites. The characteristics of plastic deformation (STZ activation and dislocations emission) of the nanolaminate composites are observed during nanoindentation loading and unloading. The main findings are as follows:

(1) The load–displacement curves of the nanolaminate composites showed small fluctuations in the amorphous layer, which are related to STZ activation, whereas larger fluctuations occur in the crystalline layer, which is related to dislocation emission. The force value increases with the increasing loading rate and the tip radius of the indenter.

(2) The local STZ activation occurred during loading, and the number of STZs increased with an increase of the indentation depth in the amorphous layer. These activated STZs were mostly concentrated around the indenter. When the indenter penetrated the crystalline layer, dislocations emitted from the amorphous/crystalline interface. They were localized around the indenter, and their number increased with increasing indentation depth. During unloading, the overall number of STZs and dislocations decreased, but also new STZs activated, and dislocations nucleated.

(3) During loading, high stresses were rather concentrated around the indenter than at other locations in the amorphous layer. When the indenter penetrated the crystalline layer, the high stresses were gradually dispersed in the amorphous layer, but the local stresses were still concentrated around the indenter in the crystalline layer. During unloading, the high stresses were dispersed in the amorphous layer, and the stressed in the amorphous layer were larger than in the crystalline layer.

## Data Availability

Data sharing is not applicable to this article.
